# Floral volatile benzenoids/phenylpropanoids: biosynthetic pathway, regulation and ecological value

**DOI:** 10.1093/hr/uhae220

**Published:** 2024-08-12

**Authors:** Mengwen Lv, Ling Zhang, Yizhou Wang, Linlin Ma, Yong Yang, Xian Zhou, Liangsheng Wang, Xiaonan Yu, Shanshan Li

**Affiliations:** School of Landscape Architecture, Beijing Forestry University, Beijing Key Laboratory of Ornamental Plants Germplasm Innovation and Molecular Breeding, National Engineering Research Center for Floriculture, Beijing 100083, China; State Key Laboratory of Plant Diversity and Specialty Crops, Institute of Botany, Chinese Academy of Sciences, Beijing 100093, China; China National Botanical Garden, Beijing 100093, China; State Key Laboratory of Plant Diversity and Specialty Crops, Institute of Botany, Chinese Academy of Sciences, Beijing 100093, China; China National Botanical Garden, Beijing 100093, China; University of Chinese Academy of Sciences, Beijing 100049, China; State Key Laboratory of Plant Diversity and Specialty Crops, Institute of Botany, Chinese Academy of Sciences, Beijing 100093, China; China National Botanical Garden, Beijing 100093, China; University of Chinese Academy of Sciences, Beijing 100049, China; State Key Laboratory of Plant Diversity and Specialty Crops, Institute of Botany, Chinese Academy of Sciences, Beijing 100093, China; China National Botanical Garden, Beijing 100093, China; University of Chinese Academy of Sciences, Beijing 100049, China; State Key Laboratory of Plant Diversity and Specialty Crops, Institute of Botany, Chinese Academy of Sciences, Beijing 100093, China; China National Botanical Garden, Beijing 100093, China; State Key Laboratory of Plant Diversity and Specialty Crops, Institute of Botany, Chinese Academy of Sciences, Beijing 100093, China; China National Botanical Garden, Beijing 100093, China; University of Chinese Academy of Sciences, Beijing 100049, China; State Key Laboratory of Plant Diversity and Specialty Crops, Institute of Botany, Chinese Academy of Sciences, Beijing 100093, China; China National Botanical Garden, Beijing 100093, China; University of Chinese Academy of Sciences, Beijing 100049, China; School of Landscape Architecture, Beijing Forestry University, Beijing Key Laboratory of Ornamental Plants Germplasm Innovation and Molecular Breeding, National Engineering Research Center for Floriculture, Beijing 100083, China; State Key Laboratory of Plant Diversity and Specialty Crops, Institute of Botany, Chinese Academy of Sciences, Beijing 100093, China; China National Botanical Garden, Beijing 100093, China; University of Chinese Academy of Sciences, Beijing 100049, China

## Abstract

Benzenoids/phenylpropanoids, the second most diverse group of plant volatiles, exhibit significant structural diversity and play crucial roles in attracting pollinators and protecting against pathogens, insects, and herbivores. This review summarizes their complex biosynthetic pathways and regulatory mechanisms, highlighting their links to plant growth, development, hormone levels, circadian rhythms, and flower coloration. External factors like light, humidity, and temperature also influence their biosynthesis. Their ecological value is discussed, offering insights for enhancing floral scent, pollinator attraction, pest resistance, and metabolic engineering through genetic modification.

## Introduction

**Figure 1:**
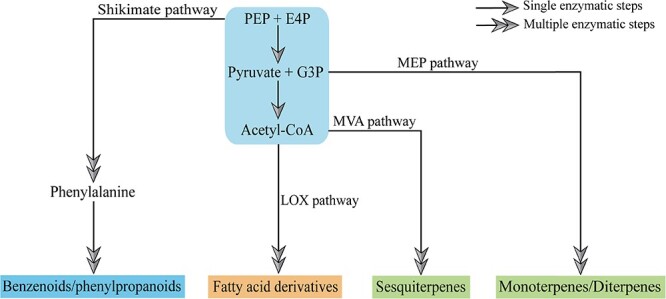
Biosynthetic pathways of plant volatile compounds. Plant VOCs are divided into three main categories, of which terpenoids, including monoterpenes, diterpenes, and sesquiterpenes, are synthesized via the methylerythritol phosphate (MEP) and mevalonic acid (MVA) pathways; fatty acid derivatives originate from the lipoxygenase (LOX) pathway; benzenoids/phenylpropanoids (BPs) are derived from the shikimate pathway. PEP, phosphoenolpyruvic acid; E4P, erythrose 4-phosphate; G3P, glyceraldehyde-3-phosphate.

Floral scent, a blend of low molecular weight, high vapor pressure volatile organic compounds (VOCs), attracts pollinators and repels insects and pathogens. Additionally, it enhances the aesthetic and economic value of ornamental plants, with applications in perfumes, cosmetics, food spices, and medicine [1]. VOCs are categorized into terpenoids, benzenoids/phenylpropanoids (BPs), and fatty acid derivatives based on their biosynthetic pathways (Fig. 1) [1, 2]. Terpenoids, including monoterpenes, diterpenes, and sesquiterpenes, are synthesized via the methylerythritol phosphate (MEP) and mevalonic acid (MVA) pathways. Fatty acid derivatives originate from the lipoxygenase (LOX) pathway, while BPs derive from the shikimate pathway (Fig. 1) [1, 2]. Extensively studied in both herbaceous and woody plants, BPs are the focus of this review, which explores their biosynthetic pathways, regulatory mechanisms, and ecological roles. This research could inform strategies for breeding plant varieties with enhanced fragrance, benefiting the fragrance industry and horticulture.

## Biosynthetic pathways of benzenoids/phenylpropanoids

The distribution of BPs in plants is extensive, BPs constituting a significant component of floral scent. They are present in many fragrant plant species, encompassing herbaceous flowers like *Petunia hybrida*, *Paeonia lactiflora*, *Clarkia breweri*, *Antirrhinum majus*, and woody flowers such as *Rosa*, *Paeonia suffruticosa*, *Prunus mume*, *Plumeria rubra*, and *Jasminum sambac* (Table 1) [3, 4, 6, 8–10, 12–14]. *Petunia hybrida*, *Rosa* spp., and *Prunus mume* are especially noted for their distinctive fragrances, largely due to BPs. The range of BPs includes compounds like benzaldehyde, phenylacetaldehyde, 2-phenylethanol, isoeugenol, eugenol, methyl benzoate, benzyl benzoate, benzyl acetate, methyl salicylate, 3,5-dimethoxytoluene, 1,3,5-trimethoxybenzene, and 1,4-dimethoxybenzene, which are common in plant floral scents (Table 1).

**Figure 2:**
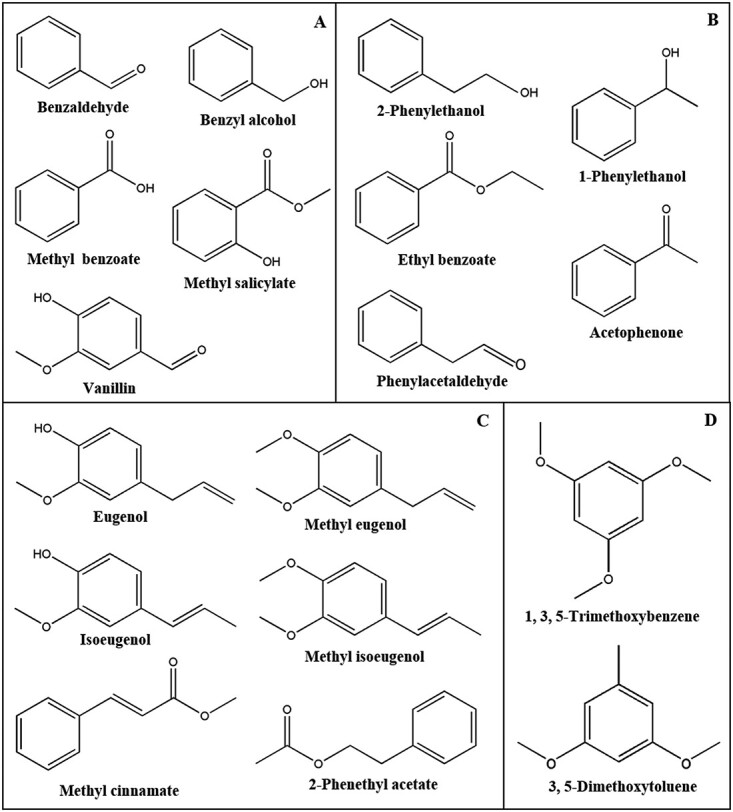
Common BPs of plant floral volatiles. According to structure and biosynthetic pathways, BPs can be roughly divided into (**A**) benzenoids (C6–C1), such as benzyl alcohol, benzaldehyde, etc.; (**B**) phenylpropanoid-related compounds (C6–C2), such as phenylacetaldehyde and 2-phenylethanol, etc.; (**C**) phenylpropanoids (C6–C3), such as eugenol, isoeugenol, etc.; and (**D**) polyketides, such as 1,3,5-trimethoxybenzene and 3,5-dimethoxytoluene, which are catalyzed by type III polyketide synthases (PKS III).

BPs are volatile compounds characterized by an aromatic carbon skeleton derived from phenylalanine (Fig. 2). The deamination of phenylalanine, catalyzed by phenylalanine ammonia lyase (PAL), produces *trans*-cinnamic acid (*t-*CA), the precursor for BPs. Subsequent methylation or acylation reactions, mediated by various enzymes, generate a diverse array of BPs [1, 18]. Based on their structure and biosynthetic pathways, BPs are classified into benzenoids (C6–C1), such as benzyl alcohol and benzaldehyde (Fig. 2A); phenylpropanoid-related compounds (C6–C2); such as phenylacetaldehyde and 2-phenylethanol (Fig. 2B); and phenylpropanoids (C6–C3), such as eugenol and isoeugenol (Fig. 2C) [1]. Additionally, BPs include polyketides like 1,3,5-trimethoxybenzene and 3,5-dimethoxytoluene, synthesized by type III polyketide synthases (PKS III) acting on cinnamyl-CoA and malonyl-CoA (Fig. 2D) [18].

**Table 1:** The distribution of benzenoids/phenylpropanoids in plant flowers.

**Species**	**Main BPs volatile compounds**	**Reference**
*Petunia hybrida*	Benzaldehyde, phenylacetaldehyde, methyl benzoate, phenylethanol, isoeugenol, benzyl benzoate	[3]
*Rosa*	3,5-Dimethoxytoluene, 1,3,5-trimethoxybenzene, eugenol, 2-phenylethanol, phenylacetaldehyde	[4, 5]
*Paeonia suffruticosa*	1,3,5-Trimethoxybenzene, 1,4-dimethoxybenzene, ethyl benzoate, phenylethyl alcohol	[6, 7]
*Paeonia lactiflora*	1,4-Dimethoxybenzene, phenylethyl alcohol, phenylethyl acetate	[8]
*Prunus mume*	Benzyl acetate, benzaldehyde, benzyl alcohol	[9]
*Clarkia breweri*	Eugenol, isoeugenol, methyleugenol, isomethyleugenol, benzyl acetate, benzyl benzoate, methyl salicylate, and vanillin	[10, 11]
*Plumeria rubra*	2-Phenylethanol, benzaldehyde, 2-phenylacetaldehyde, (*E/Z*)-phenylacetaldoxime, benzyl nitrile, 2-phenylnitroethane	[12]
*Antirrhinum majus*	3,5-Dimethoxytoluene, acetophenone, methyl benzoate, 2-hydroxyacetophenone, benzaldehyde, cinnamyl alcohol, ethyl benzoate, methyl cinnamate, methyl salicylate	[13]
*Jasminum sambac*	Benzyl acetate, methyl benzoate, methyl salicylate	[14]
*Lagerstroemia*	Benzaldehyde, phenylacetaldehyde, benzyl alcohol, eugenol, phenylethanol	[15]
*Camellia sinensis*	(*R*)-1-Phenylethanol, (*S*)-1-phenylethanol	[16]
*Stephanotis floribunda*	Benzyl alcohol, benzyl acetate, benzyl benzoate, eugenol, methyl benzoate, methyl salicylate, phenylethyl alcohol, 1-nitro-2-phenylethane	[17]

## Biosynthesis of phenylalanine

**Figure 3:**
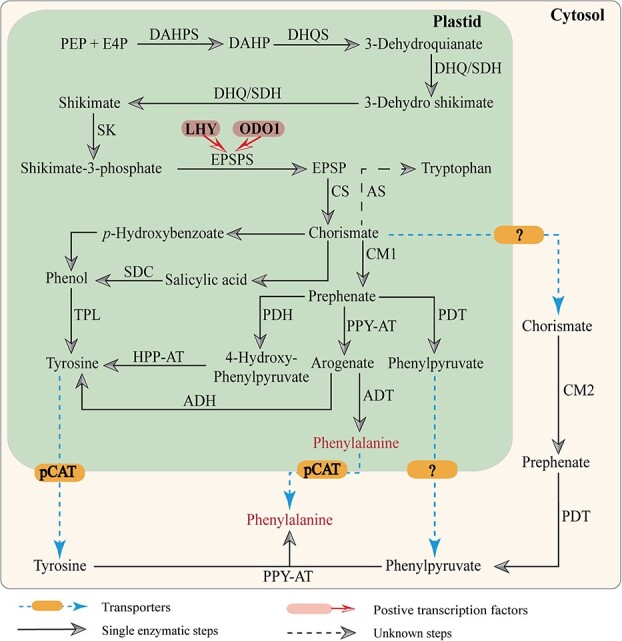
Transcriptional regulation and biosynthetic pathway of phenylalanine. Phenylalanine (Phe), the aromatic carbon skeleton of most BPs, is biosynthetically derived via the shikimic acid pathway. DAHPS, 3-deoxy-d-arabino-heptulosonate-7-phosphate synthase; DAHP, 3-deoxy-d-arabino-heptulosonate 7-phosphate synthase; DHQS, 3-dehydroquinate synthase; DHQ/SDH, 3-dehydroquinate dehydratase/shikimate 5-dehydrogenase; SK, shikimate kinase; EPSPS, 5-enolpyruvylshikimate 3-phosphate synthase; EPSP, 5-enolpyruvyl-3-shikimate phosphate; CS, chorismate synthase; AS, anthranilate synthase; CM1, chorismate mutase 1; CM2, chorismate mutase 2; SDC, salicylate decarboxylase; PDH, prephenate dehydrogenase; PDT, prephenate dehydratase; PPA-AT, prephenate aminotransferase; TPL, tyrosine phenol lyase; HPP-AT, 4-hydroxyphenylpyruvate aminotransferase; ADH, arogenate dehydrogenase; ADT, arogenate dehydratase; pCAT, plastidial cationic amino-acid transporter; ODO1, ODORANT1; LHY, LATE ELONGATED HYPOCOTYL.

Phenylalanine serves as the aromatic carbon skeleton for most BPs. Its biosynthesis primarily occurs via the shikimate pathway. The process begins with 3-deoxy-D-arabino-heptulosonate-7-phosphate synthase (DAHPS) catalyzing the conversion of phosphoenolpyruvate (PEP) from glycolysis and erythrose 4-phosphate (E4P) from the pentose phosphate pathway into 3-deoxy-d-arabino-heptulosonate-7-phosphate (DAHP). DAHPS is crucial for regulating carbon flux into the shikimate pathway (Fig. 3) [19]. Next, 3-dehydroquinate synthase (DHQS) converts DAHP into 3-dehydroquinate, which is then transformed into shikimate by 3-dehydroquinate dehydratase/shikimate 5-dehydrogenase (DHQ/SDH) (Fig. 3) [20]. Shikimate is subsequently converted into shikimate-3-phosphate by shikimate kinase (SK). 5-Enolpyruvyl-3-shikimate phosphate synthase (EPSPS) then catalyzes the transformation of shikimate-3-phosphate into 5-enolpyruvyl-3-shikimate phosphate (EPSP) [21]. Finally, chorismate synthase (CS) forms chorismate. Chorismate mutase (CM), with isoforms CM1 and CM2 located in plastids and the cytosol, respectively, converts chorismate to prephenate (Fig. 3) [22, 23]. Transamination of chorismate and prephenate leads to the synthesis of phenylalanine, tyrosine, and tryptophan, which are transported from the plastid to the cytosol via specific transporters.

In the cytosol, phenylalanine is synthesized via two pathways. In the first, tyrosine is transported from the plastid to the cytosol via the plastidial cationic amino-acid transporter (pCAT), where phenylpyruvate aminotransferase (PPY-AT) converts tyrosine and phenylpyruvate to phenylalanine. In the second pathway, chorismate is converted to prephenate by chorismate mutase (CM). Prephenate is then converted to phenylpyruvate by prephenate dehydratase (PDT) and transported to the cytosol. Alternatively, prephenate is converted to arogenate within the plastid by PPY-AT. Arogenate dehydratase (ADT) then converts arogenate to phenylalanine, which is transferred to the cytosol via pCAT (Fig. 3) [24, 25].

## Biosynthesis of benzenoids

**Figure 4:**
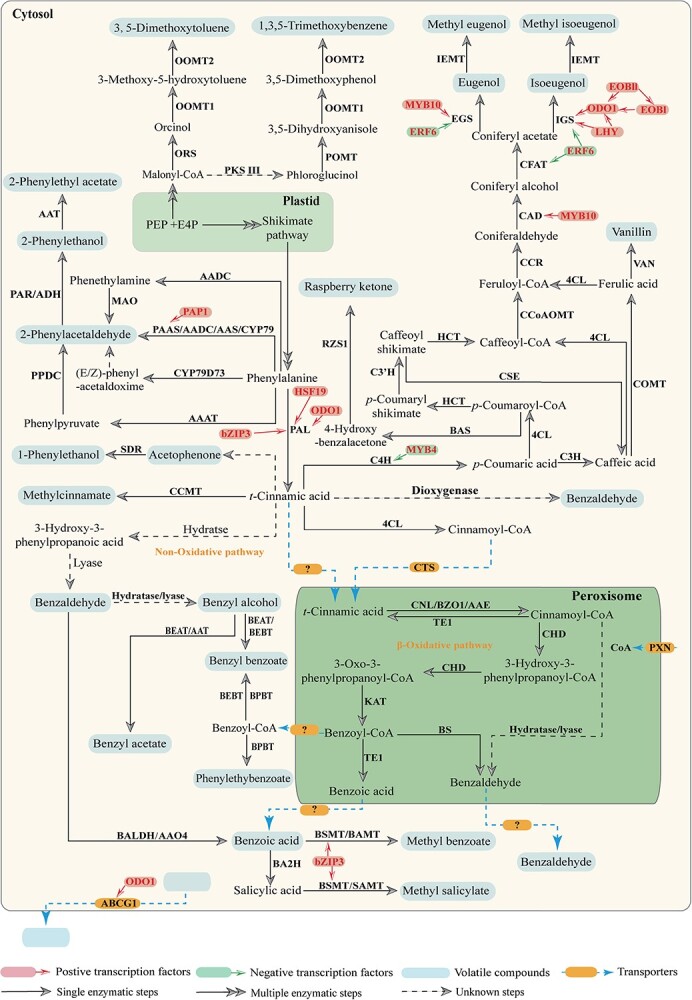
Transcriptional regulation and biosynthetic pathway of BPs. The key transcription factors, transporters, and enzymes involved in the biosynthesis pathway of BPs that have been identified are depicted in this figure. PEP, phosphoenolpyruvic acid; E4P, erythrose 4-phosphate; PAL, phenylalanine ammonia lyase; 4CL, 4-coumaroyl-CoA ligase; C4H, cinnamate-4-hydroxylase; C3H, *p*-coumarate-3-hydroxylase; C3′H, *p*-coumarate 3′-hydroxylase; BAS, benzalacetone synthase; HCT, hydroxycinnamoyl transferase; CSE, caffeoyl shikimate esterase; COMT, caffeic acid *O*-methyltransferase; CCoAOMT, caffeoyl-CoA 3-*O*-methyltransferase; CCR, cinnamoyl-CoA reductase; CAD, cinnamyl alcohol dehydrogenase; CFAT, coniferyl alcohol acetyltransferase; EGS, eugenol synthase; IGS, isoeugenol synthase; IEMT, (iso)eugenol-*O*-methyltransferase; CNL, cinnamoyl-CoA ligase; BZO1, benzoyloxyglucosinolate 1; AAE, acyl-activating enzyme; TE1, thioesterase 1; CHD, cinnamoyl-CoA hydratase-dehydrogenase; CoA, coenzyme A; KAT, 3-ketoacyl-CoA thiolase; BS, benzaldehyde synthase; BEAT, acetyl-CoA benzylalcohol acetyltransferase; BEBT, benzoyl-coenzyme A (CoA): benzyl alcohol benzoyl transferase; BPBT, benzoyl-CoA; benzylalcohol/2-phenylethanol benzoyltransferase; AAT, alcohol acetyltransferase; BALDH, benzaldehyde dehydrogenase; AAO4, arabidopsis aldehyde oxidase 4; BA2H, benzoic acid 2-hydroxylase; BSMT, benzoic acid/salicylic acid carboxyl methyltransferase; BAMT, benzoic acid carboxyl methyl transferase; SAMT, salicylic acid carboxyl methyl transferase; CCMT, cinnamic acid carboxyl methyl transferase; SDR, short chain dehydrogenase; AAAT, aromatic amino acid aminotransferase; CYP79, cytochrome P450 family 79 enzyme; AADC, aromatic amino acid decarboxylase; MAO, monoamine oxidase; PPDC, phenylpyruvate decarboxylase; PAAS, phenylacetaldehyde synthase; AAS, aromatic aldehyde synthase; PAR, phenylacetaldehyde reductase; RZS1, ketone/zingerone synthase 1; PKS III, type III polyketide synthases; ORS, orcinol synthase; POMT, phloroglucinol *O*-methyltransferase; OOMT, orcinol *O*-methyltransferase, EOBI, EMISSION OF BENZENOIDS I; EOBII, EMISSION OF BENZENOIDS II; ODO1, ODORANT1; LHY, LATE ELONGATED HYPOCOTYL; ERF, ethylene response factors; PAP1, purple acid phosphatase1; HSF19, heat shock factor 19; bZIP3, basic leucine zipper 3; PXN, peroxisomal NAD+ carrier; CTS, COMATOSE.

Benzenoids, exemplified by benzyl alcohol and benzaldehyde, typically feature a benzene ring with a one-carbon side chain (C6–C1). Their biosynthesis initiates with PAL, which deaminates phenylalanine into *t*-CA (Fig. 3). The process involves shortening the propyl side chain of *t*-CA by two carbons, accomplished through the CoA-dependent β-oxidation pathway in the peroxisome, the CoA-independent non-β-oxidation pathway in the cytosol, or a combination of both [26]. The peroxisomal NAD+ carrier (PXN) facilitates coenzyme A (CoA) transport from the cytosol to the peroxisome, supporting all CoA ligases and initiating β-oxidation (Fig. 4) [27]. The peroxisomal ATP-binding cassette transporter COMATOSE (CTS) begins the β-oxidation pathway by binding cinnamoyl-CoA, cleaving its CoA fraction during transport [28]. Cinnamoyl-CoA ligase (CNL) reactivates *t*-CA within the peroxisome, yielding cinnamoyl-CoA (Fig. 4) [29, 30]. Benzoyloxyglucosinolate 1 (BZO1) and acyl-activating enzyme (AAE) also contribute, synthesizing cinnamoyl-CoA [31, 32]. Cinnamoyl-CoA undergoes conversion to 3-hydroxy-3-phenylpropanoyl-CoA and 3-oxo-3-phenylpropanoyl-CoA by cinnamoyl-CoA hydratase-dehydrogenase (CHD) [33]. The side chain of 3-oxo-3-phenylpropanoyl-CoA is then shortened by 3-ketoacyl thiolase (KAT), forming benzoyl-CoA (Fig. 4) [34]. Benzoyl-CoA can be converted to benzoic acid or retransformed to *t*-CA via peroxisome-located thioesterase 1 (TE1) (Fig. 4) [35]. Furthermore, benzoyl-CoA produced via the β-oxidation pathway can be transported to the cytosol, where benzyl benzoate and phenylethylbenzoate are synthesized by benzoyl-CoA:benzyl alcohol/2-phenylethanol benzoyltransferase (BPBT) [26]. Silencing *BPBT* in petunia leads to the absence of benzyl benzoate and phenylethylbenzoate emissions from flowers, with other volatile compounds unaffected. Intriguingly, *BPBT*-silenced plants display notable morphological differences, such as larger flowers and leaves [26], suggesting a correlation between BPs biosynthesis and auxin.

Benzaldehyde, a crucial intermediate, plays dual roles as both a benzenoid and a key component in various chemical reactions. In the non-β-oxidation pathway, benzaldehyde is generated either by direct cleavage of the double bond in cinnamic acid by a dioxygenase enzyme or through the hydration of cinnamic acid’s double bond to form 3-hydroxy-3-phenylpropanoic acid, subsequently cleaved to yield benzaldehyde (Fig. 4). While an enzyme catalyzing benzaldehyde formation from cinnamic acid via a non-β-oxidation pathway has been identified in *Pyrus pyrifolia*, the gene encoding this enzyme remains elusive [36]. Despite potential non-β-oxidation pathways, benzaldehyde is also involved in β-oxidation pathways. In *Capsella* selfing lines, loss of benzaldehyde production was linked to the absence of cinnamate:CoA ligase 1 (CNL1), although the exact mechanism remains unclear [30]. Recent advancements have shed light on this process. Huang *et al*. [37] discovered a peroxisomal heterodimeric enzyme, benzaldehyde synthase (BS), in petunia, which converts benzoyl-CoA into benzaldehyde (Fig. 4). Subsequently, enzymes such as *Arabidopsis* aldehyde oxidase 4 (AAO4) or benzaldehyde dehydrogenase (BALDH) further metabolize benzaldehyde into benzoic acid [38, 39].

Benzaldehyde may undergo conversion to benzyl alcohol by an undisclosed alcohol dehydrogenase or be released directly as a volatile compound. Furthermore, benzyl alcohol can be derived from benzaldehyde and subsequently converted into benzyl acetate by acetyl-CoA benzylalcohol acetyltransferase (BEAT) [40]. Benzyl alcohol and benzoyl-CoA utilization by benzoyl-CoA:benzyl alcohol benzoyl transferase (BEBT) leads to benzyl benzoate production (Fig. 4) [41]. Benzoic acid, another crucial intermediate, can be methylated through benzoic acid carboxyl methyltransferase/benzoic acid/salicylic acid carboxyl methyltransferase (BAMT/BSMT), yielding methyl benzoate, or transformed into salicylic acid via benzoic acid 2-hydroxylase (BA2H). Subsequently, salicylic acid may be further methylated by BAMT/BSMT to form methyl salicylate (Fig. 4) [42–44]. *BSMT*, *SAMT*, and *BAMT*, encoding multisubstrate synthases, synthesize methyl benzoate and methyl salicylate using benzoic acid and salicylic acid as precursors. *SAMT* and *BAMT*, cloned from *C. breweri*, *Stephanotis floribunda*, and snapdragon, exhibit amino acid sequence homology with *BSMT* [42, 45, 46]. However, a key difference lies in their encoding of single-substrate enzymes. While BSMT is bifunctional, SAMT (salicylic acid carboxyl methyl transferase) and BAMT are monofunctional. These variations likely stem from plants’ evolutionary history, influencing active site diversity. Further research on this subject is warranted.

## Biosynthesis of phenylpropanoids

Phenylpropanoids, a distinct class of BPs, originate from *t*-CA. While methylcinnamate can be directly synthesized from *t*-CA via cinnamic acid carboxyl methyl transferase (CCMT), most phenylpropanoids follow a cascade of enzymatic reactions (Fig. 4) [47]. Key enzymes include cinnamate-4-hydroxylase (C4H) and 4-coumaroyl-CoA ligase (4CL). C4H, the first characterized plant P450 monooxygenase, hydroxylates *t*-CA to *p*-coumaric acid using NADPH and oxygen [48]. 4CL catalyzes CoA ester biosynthesis, including cinnamoyl-CoA, *p*-coumaroyl-CoA, caffeoyl-CoA, and feruloyl-CoA (Fig. 4) [29]. Raspberry ketone, a distinctive aromatic compound found in select plant species, derives from the reaction between *p*-coumaroyl-CoA and malonyl-CoA, mediated by benzalacetone synthase (BAS), yielding 4-hydroxybenzalacetone. Ketone/zingerone synthase 1 (RZS1) catalyzes raspberry ketone production (Fig. 4) [49]. *p*-Coumaroyl-CoA can convert to caffeoyl-CoA via hydroxycinnamoyl transferase (HCT), followed by reactions involving *p*-coumarate 3′-hydroxylase (C3′H) and caffeoyl shikimate esterase (CSE). Caffeoyl-CoA undergoes methylation catalyzed by caffeic acid *O*-methyltransferase (COMT) and caffeoyl-CoA 3-*O*-methyltransferase (CCoAOMT), producing ferulic acid and feruloyl-CoA, respectively (Fig. 4) [50–53].

Feruloyl-CoA is reduced by cinnamoyl-CoA reductase (CCR) to coniferaldehyde, which is then converted to coniferyl acetate by cinnamyl alcohol dehydrogenase (CAD) and coniferyl alcohol acetyltransferase (CFAT) (Fig. 4) [54, 55]. Eugenol and isoeugenol derive from coniferyl acetate via eugenol synthase (EGS) and isoeugenol synthase (IGS) (Fig. 4) [56]. Additionally, eugenol and isoeugenol can be methylated to methyl eugenol and methyl isoeugenol, respectively, using *S*-adenosine-l-methionine (SAM) as the methyl donor, facilitated by (iso)eugenol-*O*-methyltransferase (IEMT) (Fig. 4) [57].

## Biosynthesis of phenylpropanoid-related compounds

Phenylpropanoid-related compounds, such as 2-phenylacetaldehyde and 2-phenylethanol, compete with PAL for phenylalanine substrate (Fig. 4). The biosynthesis of these compounds varies among plant species. In tomatoes, phenylalanine is converted to 2-phenethylamine by aromatic amino acid decarboxylase (AADC), further transformed into 2-phenylacetaldehyde via monoamine oxidase (MAO), and finally converted into 2-phenylethanol by phenylacetaldehyde reductase (PAR) (Fig. 4) [58, 59]. In contrast, petunia and rose utilize a distinct pathway where phenylalanine is directly converted into 2-phenylacetaldehyde by phenylacetaldehyde synthase (PAAS), which also contributes to 2-phenylethanol production (Fig. 4) [60]. Melon follows a different route, converting phenylalanine into phenylpyruvate through aromatic amino acid aminotransferase (AAAT) before decarboxylation to form 2-phenylacetaldehyde (Fig. 4) [61].

Competing hypotheses surround the biosynthesis of 2-phenylethanol. One suggests two pathways: phenylalanine decarboxylation to produce 2-phenylethylamine, oxidized to form 2-phenylethanol, or phenylalanine conversion to phenylpyruvate, then to 2-phenylethanol (Fig. 4) [62]. Another posits three potential routes, with cytochrome P450 family 79 enzymes (CYP79) catalyzing phenylalanine conversion into an unstable intermediate, hydrolyzed to yield 2-phenylacetaldehyde. This intermediate can become 2-phenylethanol via arogenate dehydrogenase (ADH) or PAR (Fig. 4) [63]. In *Rosa* species, AAAT and phenylpyruvate decarboxylase (PPDC) are vital, facilitating reversible phenylpyruvate biosynthesis from phenylalanine, mitigating phenylpropanoid biosynthesis inhibition from high temperatures [5, 64, 65]. Petunia hosts AADC-like aromatic aldehyde synthase (AAS) and CYP79, akin to *Arabidopsis* and *Populus trichocarpa*, synthesizing phenylacetaldehyde (Fig. 4) [66, 67]. *Plumeria rubra*’s CYP79D73 influences 2-phenylacetaldehyde and 2-phenylethanol production under PAR conditions, generating (E/Z)-phenylacetaldoxime (PAOx) from phenylalanine (Fig. 4) [12]. Petunia also emits low benzyl acetate and phenylethyl acetate levels. *Rosa hybrida*’s alcohol acetyltransferase (AAT) overexpression in petunia significantly boosts compound release (Fig. 4) [68], underlining substrate availability’s significance in volatile compound biosynthesis.

The biosynthesis of BPs exhibits diverse pathways. For instance, vanillin, produced from ferulic acid by the sole enzyme vanillin synthase (VpVAN) in *Vanilla planifolia*, represents an alternative route within the phenylpropanoid biosynthetic pathway [69]. Furthermore, while acetophenone is commonly associated with enzymatic reactions from phenylalanine, it can also arise through alternative pathways. Additionally, acetophenone can be converted into 1-phenylethanol by the enzyme short-chain dehydrogenase (SDR) (Fig. 4) [16].

## Biosynthesis of polyketides

In addition to the previously mentioned categories, BPs include floral volatiles with a benzene ring structure, such as polyketides generated by polyketide synthases (PKSs) acting on cinnamoyl-CoA and malonyl-CoA [18]. These compounds, like 1,3,5-trimethoxybenzene and 3,5-dimethoxytoluene, are prominent aroma components in Chinese rose species and various modern varieties [4]. While the biosynthesis of phloroglucinol and orcinol, substrates for 1,3,5-trimethoxybenzene and 3,5-dimethoxytoluene respectively, remains incompletely understood in plants (Fig. 4), bacterial studies suggest their polyketide origin catalyzed by PKS. PKS are categorized into types I, II, and III, with type III predominantly found in plants, bacteria, and fungi. Investigations on *Pseudomonas fluorescens* have revealed six phloroglucinol synthase genes (*phlA–F*), *phlD* being crucial for phloroglucinol biosynthesis. *PhlD* catalyzes the condensation cyclization of three malonyl-CoA molecules to produce phloroglucinol. Heterologous expression of *phlD* in *Arabidopsis* yields phloroglucinol, with chloroplast localization significantly enhancing its content, suggesting foreign gene introduction as an effective method to modulate BPs profiles [70]. Similarly, orcinol synthase (ORS) in *Rhododendron dauricum*, categorized under PKS III, is responsible for synthesizing the precursor orcinol for 3,5-dimethoxytoluene [71].

In *Rosa chinensis* and *Rosa hybrida*, three genes associated with 1,3,5-trimethoxybenzene biosynthesis have been identified. The conversion of phloroglucinol into 1,3,5-trimethoxybenzene involves a sequential three-step process mediated by *O*-methyltransferases (OMTs). Specifically, phloroglucinol *O*-methyltransferase (POMT) catalyzes the transformation of phloroglucinol to 3,5-dihydroxyanisole; orcinol *O*-methyltransferase 1 (OOMT1) facilitates the conversion of 3,5-dimethoxyphenol; and OOMT2 enables the conversion of 3,5-dimethoxyphenol to yield 1,3,5-trimethoxybenzene (Fig. 4) [72, 73]. Additionally, the biosynthesis of 3,5-dimethoxytoluene from orcinol and 3-methoxy-5-hydroxytoluene can also be achieved by OOMT1 and OOMT2 (Fig. 4) [72, 74]. OOMT1 and OOMT2 exhibit distinct substrate specificities due to single amino acid polymorphisms in the phenolic substrate binding site [75]. Among the 18 species of *Rosa*, *R. chinensis* uniquely possesses both *OOMT1* and *OOMT2* genes. Evidence suggests that the evolution of *OOMT1* in *R. chinensis* likely originated from OOMT2 and may play a crucial role in floral scent production [74].

## Regulation of benzenoids/phenylpropanoids

The biosynthesis of BPs represents a central pathway for plant secondary metabolites, characterized by intricate regulation involving various transcription factors. Notably, *ODORANT1* (*ODO1*), a member of the R2R3-MYB transcription factor family, is the first identified floral scent-associated transcription factor gene in petunia. ODO1 activates the *EPSPS* gene and the ABC transporter promoter responsible for BPs transport across cell membranes, thus governing BPs biosynthesis and shikimic acid transcription (Figs 3 and 4, Table 2). Inhibition of ODO1 leads to the release of nearly all detectable BPs and affects key gene expression levels such as *DAHPS*, *EPSPS*, *PAL*, and *CM* [76, 88]. Protein-mediated active transport, as elucidated by Van Moerkercke *et al*. [87, 88], plays a crucial role in volatile channeling across the plasma membrane, with PhABCG1 facilitating BPs transport and ODO1 positively regulating their cytosolic accumulation and release (Fig. 4, Table 2) [89].

**Table 2:** Transcription factor genes involved in the biosynthesis of benzenoids/phenylpropanoids.

**Species**	**Gene families**	**Transcription factor genes**	**Regulated genes**	**References**
*Petunia hybrida*	MYB	*ODO1*	*EPSPS*, *IGS*, *ABCG1, PAL*	[76]
*Petunia hybrida*	MYB	*EOBII*	*ODO1*, *EOBI*	[77]
*Petunia hybrida*	MYB	*EOBI*	*ODO1*	[78]
*Fragaria ananassa*	MYB	*MYB10*	*EGS*	[79]
*Petunia hybrida*	HSFA	*HSF19*	*PAL*	[80]
*Petunia hybrida*	bZIP	*bZIP3*	*PAL*, *BSMT*	[81]
*Petunia hybrida*	MYB	*MYB4*	*C4H*	[82]
*Petunia hybrida*	LHY	*LHY*	*EPSPS*, *IGS*, *ODO1*	[83]
*Petunia hybrida*	AP2/ERF	*ERF6*	*IGS*, *CFAT*	[84]
*Petunia hybrida*	MYB	*PAP1*	*PAAS*	[85]
*Petunia hybrida*	MYB	*PH4*		[86]

*EMISSION OF BENZENOIDS II* (*EOBII*), a key regulatory gene in the BPs biosynthetic pathway, belongs to the R2R3-MYB transcription factor family. It governs the transcription of key enzymes and activates ODO1 and IGS promoters (Fig. 4, Table 2) [77]. Inhibiting EOBII activity markedly reduces floral scent production, including benzaldehyde, 2-phenylethanol, benzyl benzoate, and isoeugenol [77]. EMISSION OF BENZENOIDS I (EOBI), downstream of EOBII, is crucial and directly regulated by it. Silencing *EOBI* downregulates *ODO1* and genes involved in scent biosynthesis, reducing aroma emission (Fig. 4, Table 2) [78, 87]. LhODO1 from *Lilium* spp. upregulates PAL in transgenic petunia [90]. These findings underscore the complex interplay among ODO1, EOBI, and EOBII in BPs regulation.

Recent discoveries have elucidated the role of transcription factors in BPs biosynthesis. In *Fragaria ananassa*, MYB10 enhances EOBII transcription by activating *CAD* and *EGS*, thus facilitating eugenol production (Fig. 4, Table 2) [79]. Moreover, petunia’s heat shock factor 19 (HSF19) upregulates *PAL* expression (Fig. 4, Table 2) [80]. Members of the basic leucine zipper (bZIP) family, such as petunia’s *bZIP3*, bind to *PAL* and *BSMT* promoters, resulting in their upregulation (Fig. 4, Table 2) [81]. Conversely, transcription factors like MYB4 inhibit *C4H* activity, thereby regulating carbon allocation to phenylpropanoid metabolism and scent transcription (Fig. 4, Table 2) [82].

**Figure 5:**
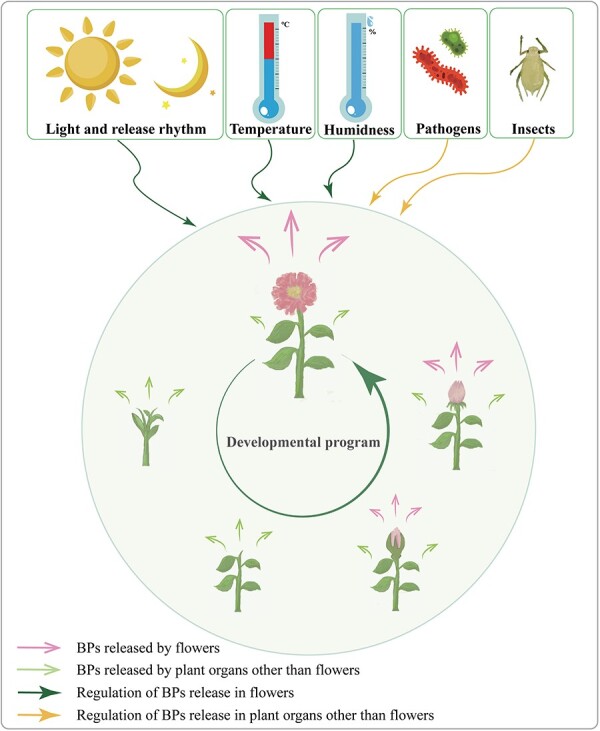
Other influencing factors involved in the regulation of BPs. Developmental stages and external factors (light, release rhythm, temperature, and humidity) also have an impact on the release of BPs in plant flowers.

Plants strategically release floral scents to attract pollinators, synchronizing with pollinator foraging patterns in a phenomenon known as release rhythm. This temporal release optimizes resource use and pollinator attraction. Snapdragon flowers, for example, emit high levels of methyl benzoate during the day, while tobacco and petunia, in which methyl benzoate is a major scent component, release it predominantly at night, reflecting the preferences of their pollinators [91, 93]. Notably, snapdragon flowers maintain this release rhythm even under continuous light or darkness, with benzoic acid accumulation and the expression of related genes (*BAMT* and *PAL*) following the release rhythm [91]. In contrast, petunia flowers exhibit strong diurnal oscillations in genes related to BPs biosynthesis (*DAHPS*, *EPSPS*, *ADT*, *CM1*, *EGS*, *IGS*, *KAT1*, and *BPBT*), but these oscillations weaken under continuous darkness and vanish in continuous light [76, 83]. This indicates that plants develop specific floral scent release rhythms influenced by internal biological clocks or external stimuli (such as light changes), thereby regulating BPs biosynthesis and release (Fig. 5). Transcription factors like ODO1, EOBI, and EOBII also show diurnal rhythms [76, 77]. The biological clock regulator LATE ELONGATED HYPOCOTYL (LHY) binds to the *ODO1* promoter, regulating evening elements (EEs) in the *EPSPS* and *IGS* promoters, and thereby controlling BPs biochemical synthesis and release (Fig. 4, Table 2) [83].

Additionally, plant hormone concentrations can significantly impact BPs biosynthesis in flowers. Ethylene response factors (ERFs), belonging to a large transcription factor family with AP2 domains, play vital roles in ethylene response pathways, stress responses, and plant development [94]. In petunia, ERF6 competes with EOBI’s c-myb domain for binding to the promoters of *CFAT*, *EGS*, and *IGS*, negatively regulating BPs biosynthesis by controlling *ODO1* expression. Ethylene treatment diminishes *BSMT* and *CFAT* expression by affecting ERF6 function, leading to reduced expression of structural genes in the BPs biosynthetic pathway (Fig. 4, Table 2) [84]. Targeted suppression of the IAA-related gene *HcIAA2* in *Hedychium coronarium* significantly increases ocimene, linalool, and methyl benzoate contents [92]. Salicylic acid (SA), functioning as both a plant hormone and a precursor for methyl salicylate production [44], directly impacts methyl salicylate release through fluctuations in SA content. External hormone treatments can trigger biochemical signals that affect plant secondary metabolism. The application of exogenous abscisic acid (ABA), jasmonic acid (JA), and methyl jasmonate (MeJA) influences the biosynthesis and release of BPs in plants [95, 96]. While JA and MeJA activate various transcription factors and are crucial to secondary metabolism, the exact mechanisms by which they regulate BPs are not yet fully understood [97].

The biosynthetic pathways of BPs overlap with those of other secondary metabolites, often competing for carbon flux. For example, phenylalanine can be converted by PAAS into 2-phenylacetaldehyde, which is then transformed into 2-phenylethanol. Alternatively, phenylalanine can be converted to *t*-CA by PAL, serving as a precursor for benzaldehyde, benzyl alcohol, or coumaric acid, and contributing to the formation of phenylpropanoids, anthocyanins, and lignins. This competition influences the color and scent of flowers [54, 56, 85, 98]. Changes in flower color and scent are typically linked to alterations in gene expression regulated by transcription factors. Increasing evidence highlights the role of regulatory factors from various families in modulating flower color and scent [98]. Transgenic petunia plants expressing the *Arabidopsis* MYB transcription factor purple acid phosphatase 1 (PAP1) display deeper coloration and significantly upregulate *PAAS* expression, resulting in a 10-fold increase in BPs content (Fig. 4, Table 2) [85]. The MYB transcription factor PH4 regulates vacuolar acidity to control flower color. Silencing *PH4* leads to significant phenylpropanoid release without affecting the expression of genes associated with phenylpropanoid biosynthesis (Fig. 4, Table 2). Therefore, researchers speculate that its correlation with floral scent release may be attributed to epidermal glycosylation of compounds [86].

**Figure 6:**
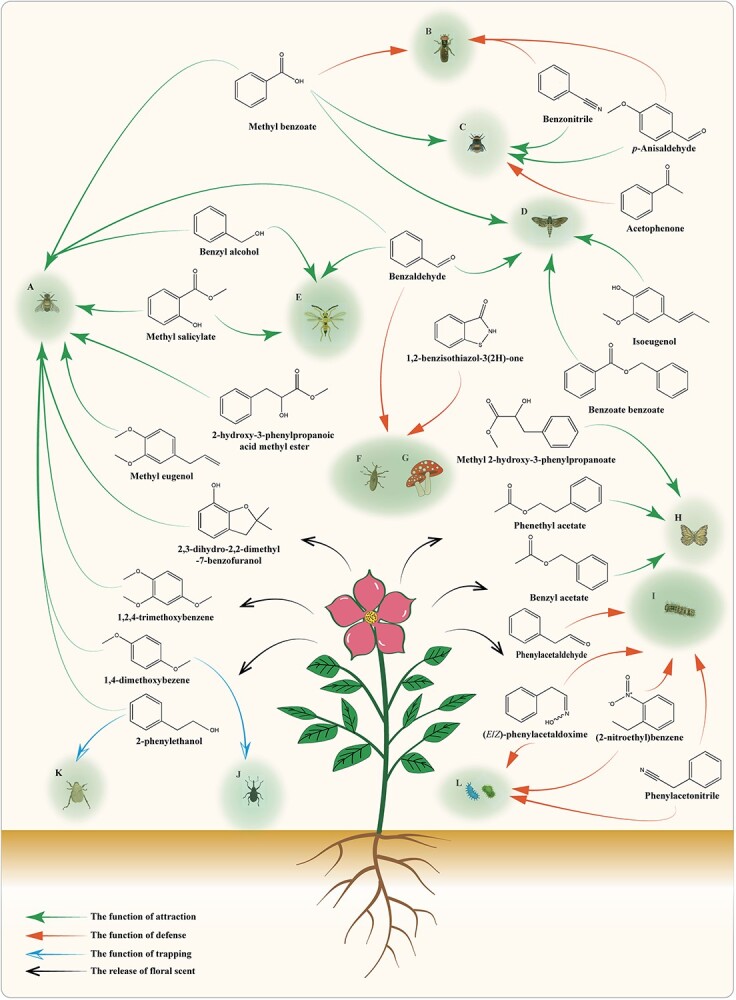
Ecological value of benzenoids/phenylpropanoids. Plant flowers release BPs, which are crucial for both luring pollinators and protecting against insects, pathogens, and herbivores. A, bees; B, hoverflies; C, bumblebees; D, moths; E, females of the rice planthopper egg parasitoid; F, weevils; G, fungi; H, butterflies; I, herbivores; J, strawberry blossom weevils; K, long-legged chafer; L, pathogens.

The biosynthesis and release of BPs in plants are influenced by various external environmental factors (Fig. 5), highlighting the intricate interplay between plant physiology and environmental cues. Factors such as light intensity, temperature, and humidity impact not only plant growth and development but also the release of BPs in plant flowers [99–101]. Additionally, the stage of plant development significantly shapes the biosynthesis and release of BPs (Fig. 5). Generally, fully opened flowers exhibit higher levels of specific BPs such as benzaldehyde, benzyl alcohol, and benzyl benzoate to attract particular pollinators [102]. Conversely, other plant tissues such as leaves and stems can continuously emit BPs or respond to pathogens and insect damage by releasing them (Fig. 5) [103, 104].

## Ecological value of benzenoids/phenylpropanoids

BPs play crucial ecological roles in plants, acting as pivotal mediators of plant-pollinator interactions, defense mechanisms against herbivores and pathogens, and contributors to stress resistance. BPs volatiles serve as vital cues for attracting specific pollinators; for instance, snapdragon emits VOCs mainly composed of BPs and terpenoids to attract a limited range of bees (Fig. 6) [105]. Daffodils, with diverse terpenoids and methyl 2-hydroxy-3-phenylpropionate as the main VOCs, are primarily pollinated by bees, while benzenoids and monoterpenes act as the main attractants for butterflies (Fig. 6) [106]. *Brassica rapa* is predominantly pollinated by bumblebees and hoverflies. After 11 generations of cross-pollination, it was observed that bumblebees preferred the enhancement of benzenoids composed mainly of anisaldehyde and benzonitrile through methyl benzoate treatment, whereas plants pollinated by hoverflies exhibited a declining trend in VOCs production [120]. Furthermore, bumblebees have been found to display distinct defensive behavior against acetophenone (Fig. 6) [107]. Additionally, researchers have identified numerous benzenoids in natural honey, such as methyl eugenol, furanols, and benzaldehyde [108]. Apple flowers attract bees for pollination by releasing floral substances containing benzyl alcohol [109]. Pumpkin flowers primarily attract insects for pollination through the release of 1,4-dimethoxybenzene and 1,2,4-trimethoxybenzene, with nectar serving as the main site for their release (Fig. 6) [121]. The release of floral scent in many plants follows a circadian rhythm that may be linked to insect activity. Honeybee-pollinated snapdragon flowers exhibit a peak in methyl benzoate emission during daylight hours [42]. Moth-pollinated petunias release floral scent such as benzaldehyde, methyl benzoate, and isoeugenol at night [3]. Methyl benzoate is one of the most abundant aromatic compounds found in snapdragon flowers, and its emission shows a clear circadian rhythm with highest levels during the daytime, aligning with bumblebee foraging patterns [91].

Besides attracting pollinators, BPs metabolites also serve as chemical barriers for plants to defend against flower insects, pathogens, and herbivores. Additionally, they play a crucial role in enhancing plant stress resistance and pest control. Phenylacetaldehyde, for instance, plays a role in defending against herbivore attacks (Fig. 6) [67]. 2-Phenylethanol, a volatile component of *Rosa* spp., has a significant trapping effect on the long-legged chafer (Fig. 6) [110]. Soybeans can synthesize phenylethanol *in vivo* after exposure to microorganisms, which can influence the community composition and abundance of arthropods around plants within an extended range of up to 8 m away [111]. Mao *et al*. [112] discovered that benzyl alcohol, benzaldehyde, and methyl salicylate present in rice VOCs attract female rice planthopper egg parasitoids (Fig. 6). A lure made with equal proportions of rice BPs can attract female rice planthopper egg parasitoids effectively, contributing to biological control measures for rice cultivation. Benzaldehyde and 1,2-benzisothiazol-3(2H)-one have been found to inhibit the growth of weevils and fungi in the surrounding soil area, providing more benefits for plant growth and development (Fig. 6) [113–115]. The addition of wild strawberry flowers’ dominant volatile compound, 1,4-dimethoxybenzene, as an aggregation pheromone increased the number of trapped strawberry blossom weevils by over two times compared with using the aggregation pheromone alone (Fig. 6) [116]. Distinctive N-containing volatiles such as phenylacetonitrile (PAN), 2-nitroethylbenzene (NEB), and (*E/Z*)-phenylacetaldoxime (PAOx) have been strongly implicated in defense against herbivores and pathogens [117–119].

## Concluding remarks and future perspectives

Floral scent, a pivotal plant trait, has garnered extensive attention, especially concerning BPs, the second major category of VOCs in floral scent. These compounds, crucial secondary metabolites, boast diverse arrays and intricate biosynthetic pathways. Recent advances have laid the foundation for breeding and transgenic engineering to manipulate plant floral scents. In contrast to terpenoids, research on BPs is largely limited to model plants like *Petunia hybrida* and faces significant constraints. Therefore, it is crucial to expand research to include a wider range of plant species and explore the diversity of BPs biosynthesis pathways across various flowers. Special attention should also be given to enzymes involved in BPs synthesis, particularly those facilitating the production of benzaldehyde and acetophenone. Understanding the varied biosynthetic pathways of compounds like 2-phenylethanol and 2-phenyletaldehyde is crucial. Moreover, unexplored catalytic mechanisms await discovery for multifunctional enzymes like CHD, IEMT, and BSMT. Despite progress in VOCs synthesis understanding, inter-organelle transport processes and cellular release mechanisms remain inadequately elucidated, necessitating comprehensive exploration.

The biosynthesis and release of plant BPs are intricately regulated by a complex interplay of transcriptional regulators and external factors, enabling plants to adapt to diverse environmental conditions. However, the limited number of identified BPs-related transcription factors calls for further in-depth investigation. Notably, transcription factors EOBI, EOBII, and ODOI exhibit intricate relationships and synergistically regulate BPs biosynthesis, warranting special attention. Additionally, factors like ERF6, LHY, PAP1, and PH4 are implicated in various processes, including hormone synthesis and the circadian rhythm, suggesting co-regulatory relationships between BPs biosynthesis and plant growth and development that merit exploration. Understanding the mutual regulation mechanisms of flower coloration and floral scent is significant for attracting pollinators. Furthermore, environmental cues such as light intensity, temperature, and humidity can influence BPs release, but the molecular mechanisms underlying these signals remain unexplored, necessitating further studies for elucidation.

The role of BPs in ecological interactions between plants and pollinators, as well as in plant defense against insects, pathogens, and herbivores, is crucial for promoting resilience and adaptability in diverse ecosystems and for plant reproduction and evolution. While the diversity of BPs is extensive, current research on their ecological value only scratches the surface, exploring a limited range of substances. There is a multitude of unexplored ecological effects associated with BPs that warrant further investigation. Moreover, exploring hybridization, transgenic techniques, or exogenous spraying of specific BPs can modify the VOCs composition in plant flowers, attracting targeted pollinators and enhancing natural pollination efficiency. Additionally, leveraging the defensive and trapping characteristics of BPs can facilitate the development of eco-friendly pesticides and green traps that minimize damage to both plants and the environment.

## Data Availability

All data have been included in the article.
